# *Akkermansia muciniphila* and its culture supernatant ameliorate colitis in interleukin-10 knockout mice via gut barrier and immune modulation

**DOI:** 10.3389/fimmu.2025.1693007

**Published:** 2025-11-17

**Authors:** Mingshan Jiang, Yongbin Jia, Chunxiang Ma, Zhen Zeng, Yan Li, Lili Li, Hao Lin, Jiangmei Pang, Jiaxin Li, Jingjing Chen, Xi Li, Kexin Chen, Hong Zhang, Yu Gan, Zou Xiang, Linlin Chen, Hu Zhang

**Affiliations:** 1 Department of Gastroenterology, West China Hospital, Sichuan University, Chengdu, Sichuan, China; 2 Centre for Inflammatory Bowel Disease, West China Hospital, Sichuan University, Chengdu, China; 3 Laboratory of Inflammatory Bowel Disease, Institute of Immunology and Inflammation, Frontiers Science Center for Disease-Related Molecular Network, West China Hospital, Sichuan University, Chengdu, China; 4 The First Department of the Digestive Disease Center, Suining Central Hospital, Chengdu, China; 5 Department of Health Technology and Informatics, Hong Kong Polytechnic University, Hong Kong, Hong Kong SAR, China; 6 Department of Gastroenterology, Suining Central Hospital, Suining, Sichuan, China

**Keywords:** *Akkermansia muciniphila*, colitis, IL-10, gut barrier, gut microbiota

## Abstract

**Background:**

*Akkermansia muciniphila* (*A. muciniphila*) is a mucin-degrading commensal bacterium with established roles in maintaining intestinal homeostasis and modulating host immune responses. However, its therapeutic potential and mechanisms in chronic spontaneous colitis remain incompletely defined. Interleukin-10 knockout (IL-10^−^/^−^) mice are a well-established model of spontaneous colitis due to impaired anti-inflammatory cytokine signaling and disrupted immune regulation. In this study, we aimed to determine whether *A. muciniphila* and its culture-derived supernatant could ameliorate intestinal inflammation in IL-10^−^/^−^ mice.

**Methods:**

We administered *A. muciniphila* or its culture supernatant (*A. muciniphila* supernatant [A.m-SN]) to IL-10^−^/^−^ mice and monitored disease progression over 30 days. Clinical assessments included body weight, disease activity index (DAI), colon length, histopathology, and endoscopy. Tight junction proteins (zonula occludens-1 [ZO-1] and Occludin) and colonic messenger ribonucleic acid levels of pro- and anti-inflammatory cytokines were evaluated. Gut microbiota composition was assessed using 16S ribosomal deoxyribonucleic acid sequencing, and untargeted metabolomic profiling was performed on the culture medium to identify potential anti-inflammatory components.

**Results:**

Mice treated with *A. muciniphila* or A.m-SN showed significantly reduced colitis severity, including reduced body weight loss, lower DAI, preserved colon length, and improved histological scores. Immunofluorescence revealed increased expression of ZO-1 and Occludin in colonic tissues. Pro-inflammatory cytokines (tumor necrosis factor-α, IL-6, IL-17, IL-1β, and interferon-γ) were downregulated, while anti-inflammatory cytokines (IL-4, transforming growth factor-β, and IL-22) were upregulated in treated groups. Gut microbiota analysis showed partial restoration of microbial diversity and structure. Metabolomic profiling identified distinct signatures in A.m-SN, with bioactive molecules such as adenosine and tryptophan derivatives implicated in the anti-inflammatory effects.

**Conclusion:**

The culture supernatant of *A. muciniphila* alleviated spontaneous colitis in IL-10^−^/^−^ mice by suppressing pro-inflammatory cytokines, enhancing anti-inflammatory mediators, and strengthening epithelial barrier function. Metabolomic profiling identified candidate bioactive components, including adenosine and tryptophan derivatives, supporting its potential as a postbiotic intervention for inflammatory bowel disease.

## 1 Introduction

Interleukin-10 knockout (IL-10^−^/^−^) mice are a widely accepted model for studying the pathogenesis and treatment of inflammatory bowel disease (IBD), particularly Crohn’s disease (1–3). In the absence of the anti-inflammatory cytokine IL-10, these mice spontaneously develop chronic enterocolitis, typically between 7 and 15 weeks of age under specific pathogen-free (SPF) or conventional housing conditions (4–6). The inflammation is driven by immune responses against commensal microbiota and is characterized by epithelial barrier disruption, immune cell infiltration, and persistent mucosal inflammation (7, 8). Given the microbial dependence of disease progression, IL-10^−^/^−^ mice provide a valuable model for evaluating how microbial interventions can modulate gastrointestinal pathology in a genetically susceptible host.

The intestinal microbiome has emerged as a crucial regulator of immune homeostasis and mucosal integrity (9–12). Perturbations in microbial composition and function, known as dysbiosis, are closely linked to IBD pathogenesis (13–17). Among gut commensals, *Akkermansia muciniphila* (*A. muciniphila*) has attracted considerable attention as a potential next-generation probiotic (18). This mucin-degrading bacterium strengthens epithelial barrier function, regulates mucosal immunity, and attenuates inflammation in multiple disease models (19–22). Recent evidence indicates that the functional effects of *A. muciniphila* are strain- and preparation-dependent. Distinct isolates and formulations (live, pasteurized, or cell-free derivatives) show heterogeneous capacities to influence mucin utilization, metabolite production, epithelial barrier function, and host immune responses, which may explain variable anti-inflammatory efficacy across experimental models (23). A randomized clinical trial reported that pasteurized *A. muciniphila* supplementation improved metabolic and immune health in humans, with enhanced mucosal barrier function and reduced inflammatory markers (24). In murine models, pasteurized *A. muciniphila* and a purified outer membrane protein (Amuc_1100) alleviated colitis by suppressing pro-inflammatory cytokines, modulating cluster of differentiation (CD)8^+^ T cells and macrophages, and reducing colitis-associated tumorigenesis through enhanced cytotoxic T lymphocyte activity (25). Collectively, these findings highlight multiple mechanisms by which *A. muciniphila* and its derivatives may promote mucosal barrier integrity, immune modulation, and inflammatory regulation, all directly relevant to their therapeutic potential in IBD. These findings suggest a promising role for this bacterium as both a therapeutic candidate and microbial biomarker. However, its therapeutic feasibility and mechanisms of action in genetically susceptible colitis models, such as IL-10^−^/^−^ mice, remain poorly defined. Moreover, the contribution of its secreted culture supernatant to these effects has yet to be fully clarified.

In this study, we aimed to determine whether *A. muciniphila* and its culture-derived supernatant could ameliorate intestinal inflammation in IL-10^−^/^−^ mice. We further explored how *A. muciniphila* exerts its anti-inflammatory effects, focusing on its ability to enhance the intestinal epithelial barrier by upregulating tight junction proteins and to improve gut microbiota dysbiosis. In addition, we conducted untargeted metabolomic analysis of *A. muciniphila*–conditioned medium to identify candidate components of the culture supernatant potentially responsible for its protective effects. These findings are expected to provide novel insights into microbial-based therapeutic strategies for IBD.

## 2 Materials and methods

### 2.1 Animal models and treatment protocols

Male IL-10^−^/^−^ mice (4–5 weeks old) and wild-type (WT) C57BL/6J mice (4–5 weeks old) were reared in a SPF facility at West China Hospital. IL-10^−^/^−^ mice were obtained from the Shanghai Model Organisms Center, and C57BL/6J mice were obtained from Jiangshu GemPharmatech. All experimental protocols were approved by the West China Hospital Animal Ethics Committee (Approval No. 20230226057).

To confirm that the oral gavage procedure itself did not confound disease outcomes, preliminary experiments were conducted in which IL-10^−^/^−^ mice were divided into two groups: one group received no treatment (IL-10^−^/^−^), and the other received daily gavage with vehicle (200 μL phosphate-buffered saline [PBS]) alone (IL-10^−^/^−^ + PBS). These results showed no significant differences in body weight, disease activity index (DAI), histological activity index (HAI), colon length, or murine endoscopic index of colitis severity (MEICS)between the two groups (**Supplementary Figure 1**). Based on these findings, a separate gavage-only control group was not included in the main study. Instead, an uninoculated culture medium (UM) group, which underwent identical preparation and handling as the *A. muciniphila*–inoculated medium but without bacterial inoculation, was used as the functional vehicle control.

Mice were randomly assigned to five groups: (1) IL-10^−^/^−^, receiving no treatment (n = 4); (2) IL-10^−^/^−^ + UM, administered 200 μl of uninoculated culture medium by oral gavage daily (n = 4); (3) IL-10^−^/^−^ + *Akkermansia muciniphila* (A.m), administered *A. muciniphila* (1 × 10^9^ colony-forming units in 200 μl PBS) by oral gavage daily (n = 4); (4) IL-10^−^/^−^ + *A. muciniphila* supernatant [A.m-SN], administered 200 μl of *A. muciniphila* culture supernatant containing its metabolic products by oral gavage daily (n = 4); and (5) WT mice, receiving no treatment (n = 4). The intervention lasted for 30 days, during which body weight, stool consistency, and fecal occult blood were recorded every other day. On day 30, all mice were euthanized for further analyses. The extent of intestinal inflammation was assessed by DAI, HAI (26), and MEICS (27). For downstream 16S rDNA sequencing analyses, fecal microbiota sequencing was performed on IL-10^−^/^−^ (n = 14), IL-10^−^/^−^ + UM (n = 22), IL-10^−^/^−^ + A.m (n = 25), and IL-10^−^/^−^ + A.m-SN (n = 13); metabolomics analysis included n = 5 samples per group.

### 2.2 Immunofluorescence

Occludin (Proteintech, China) and ZO-1 (Proteintech, China) were detected in colonic biopsy tissues by immunofluorescence staining, and nuclei were visualized with DAPI staining. Fluorescent images were registered via a Nikon fluorescence microscope (Nikon, Japan) or a VS200 imaging system (Olympus, Japan) along with subsequently analyzed with ImageJ software.

### 2.3 Fecal genomic DNA extraction

Mouse fecal DNA was isolated with the TIANamp Stool Kit (TIANGEN, China) following the supplier’s protocol.

### 2.4 Bacterial cultivation and preparation of culture supernatant

*Akkermansia muciniphila* (ATCC BAA-835™, ATCC, USA) was cultured in an anaerobic chamber (Gene Science, China) under strictly anaerobic conditions (5% H_2_, 5% CO_2_, and 90% N_2_) at 37 °C for 48 h. The growth medium consisted of brain heart infusion (BHI; Hopebio, China) supplemented with 0.05% (w/v) L-cysteine (Solarbio, China) as a reducing agent and 0.5% (w/v) porcine gastric mucin (Yuanye Bio-Technology, China) as a carbon and nitrogen source. At the end of incubation, bacterial cells were harvested by centrifugation at 10,000 × g for 10 min at 4°C, washed twice with PBS and resuspended in PBS. The cell density was determined spectrophotometrically at 600 nm (OD_600_), and the suspension was adjusted to 1.0 × 10^9^ CFU/mL. Aliquots were stored at –80°C until use.

For preparation of the culture supernatant, the same batch of sterile medium was divided into two equal portions before incubation. One portion was inoculated with *A. muciniphila*, while the other portion was left uninoculated to serve as a negative control. After 48h of culture under the same anaerobic conditions, both the inoculated and uninoculated media were subjected directly to high-speed centrifugation at 20,000 × g for 20 min at 4°C using a floor-standing centrifuge (Optima XPN-100, Beckman). The resulting supernatants were carefully collected, aliquoted, and stored at –80°C until use. These preparations were used either for oral gavage in mice or for subsequent metabolomic analysis. For animal experiments, the supernatant from the uninoculated medium was referred to as UM (uninoculated medium) and used as the vehicle control. For metabolomic analysis, the supernatant from *A. muciniphila*-inoculated medium was designated A.m-SN, while the supernatant from uninoculated medium was designated Ctrl, serving as the corresponding reference group for metabolite comparisons.

### 2.5 Untargeted metabolomics of culture supernatants

Culture supernatant samples (1 mL each) from two groups:A.m-SN (culture medium inoculated with *A. muciniphila*) and Ctrl (uninoculated culture medium) were collected (n = 5 per group). Samples were lyophilized and reconstituted in 100 µL of 80% methanol (LC–MS grade). After vortexing and incubation on ice for 5 min, the samples were centrifuged at 15,000 g for 15 min at 4°C. The supernatants were diluted with LC–MS–grade water to a final methanol concentration of 53%, followed by a second centrifugation (15,000 g, 4°C, 15 min). The clarified extracts were collected for LC–MS analysis. Quality control (QC) samples were prepared by pooling equal aliquots from each experimental sample, and blank samples (53% methanol) were processed in parallel using the same procedure.

Metabolomic profiling was performed on a high-resolution Q Exactive Plus Orbitrap mass spectrometer (Thermo Fisher, USA) coupled to a UHPLC system. Data were acquired in both positive and negative electrospray ionization (ESI) modes with a scan range of m/z 100–1500. ESI source parameters were as follows: spray voltage, 3.5 kV; sheath gas flow rate, 35 psi; auxiliary gas flow rate, 10 L/min; capillary temperature, 320°C; S-lens RF level, 60; auxiliary gas heater temperature, 350°C. MS/MS spectra were collected using data-dependent acquisition (DDA).

Raw data were processed with Progenesis QI v2.2 (Waters, UK) for peak detection, alignment, and normalization, and statistical analysis was conducted in R v3.4.0. Metabolite identification was based on accurate mass and MS/MS spectral matching against MoNA and HMDB databases. Differential culture supernatant was screened using both univariate (Wilcoxon test, p < 0.1) and multivariate (PLS-DA, VIP > 1) criteria.

### 2.6 Western blot analysis

Colon tissues from IL-10^−^/^−^, IL-10^−^/^−^ + UM, IL-10^−^/^−^ + A.m, and IL-10^−^/^−^ + A.m-SN groups were homogenized in RIPA lysis buffer (Thermo Fisher, USA) supplemented with protease and phosphatase inhibitor cocktail (Roche, Switzerland). Protein concentrations were measured using the BCA assay (Thermo Fisher, USA).

Equal amounts of protein (≈25 µg per lane) were loaded onto 10% SDS–PAGE gels. Electrophoresis was performed at 80 V for 30 min followed by 120 V until the marker bands were well separated. Proteins were then transferred to PVDF membranes (Millipore, USA), pre-activated with methanol, using a semi-dry sandwich system (sponge–filter paper–gel–PVDF–filter paper–sponge) at a constant current of 270 mA for 105 min.

Membranes were blocked in 5% non-fat milk in TBST for at least 1 h at room temperature, and then incubated overnight at 4°C with primary antibodies: anti-ZO-1 (1:5000; Proteintech, China), anti-occludin (1:5000; Proteintech, China), and anti-GAPDH (1:2000; CST, USA). After washing three times in TBST (15 min each), membranes were incubated with HRP-conjugated secondary antibodies (1:10,000; CST, USA) for 1 h at room temperature.

Protein bands were visualized using enhanced chemiluminescence (ECL; Thermo Fisher, USA), and images were captured with a chemiluminescence imaging system. Band intensity was quantified using ImageJ software (NIH, USA), and target protein expression was normalized to β-actin.

### 2.7 RNA preparation and real-time quantitative PCR analysis

Rectal RNA was isolated with the Tissue Total RNA Kit (Vazyme, China) following the supplier’s protocol. cDNA synthesis was carried out using HiScript III RT SuperMix (Vazyme, China), and gene expression was quantified on a QuantStudio 7 Pro qPCR system (Thermo Fisher, USA).

### 2.8 16S rDNA sequencing

For microbial profiling, 16S rRNA V3–V4 fragments were amplified from DNA with 515F/806R primers using Phusion^®^ High-Fidelity PCR Master Mix (NEB, USA). Sequencing was performed on Illumina NovaSeq, yielding paired-end reads of 250 bp. In reference to index tags and PCR primer reads, raw sequencing data were demultiplexed to assign reads to their respective samples. Following trimming of index tags and primer sequences, paired-end reads were merged using FLASH (v1.2.7; http://ccb.jhu.edu/software/FLASH/) (28) to generate raw tags (Raw Tags). The Raw Tags were subjected to stringent quality filtering (29) to obtain high-quality tags (Clean Tags), following the quality control pipeline in QIIME (v1.9.1; http://qiime.org/scripts/split_libraries_fastq.html) (30). Specifically: (a) Raw Tags were truncated at the first low-quality base position where the number of consecutive bases with quality scores ≤ 19 reached the set length threshold (default: 3 bases); and (b) Reads with <75% continuous high-quality bases were removed. Chimeric sequences were identified by aligning tags to the reference database using VSEARCH (https://github.com/torognes/vsearch/) (31) and subsequently removed to yield the final set of effective tags (Effective Tags). All Effective Tags from all samples were clustered into operational taxonomic units (OTUs) at 97% sequence identity using UPARSE (v7.0.1001; http://www.drive5.com/uparse/) (32). For each OTU, the most abundant sequence was designated as the representative sequence. Representative OTU sequences were assigned taxonomy using Mothur with the SILVA 132 database (confidence 0.8–1). MUSCLE v3.8.31 was used for phylogenetic alignment. Data were rarefied to the lowest sequencing depth before diversity analyses (33–35).

### 2.9 Statistical analysis

Statistical analyses were performed using GraphPad Prism 9 (GraphPad Software, CA, USA). Data are presented as mean ± standard deviation. For two-group comparisons, either Student’s t-test or Mann–Whitney U test was used depending on data distribution. For comparisons among multiple groups, one-way analysis of variance followed by Tukey’s multiple comparison test was applied. A *p-*value < 0.05 was considered statistically significant.

## 3 Results

### 3.1 *A.muciniphila* and its culture supernatant alleviate intestinal inflammation in IL-10 knockout mice

**Figure 1:**
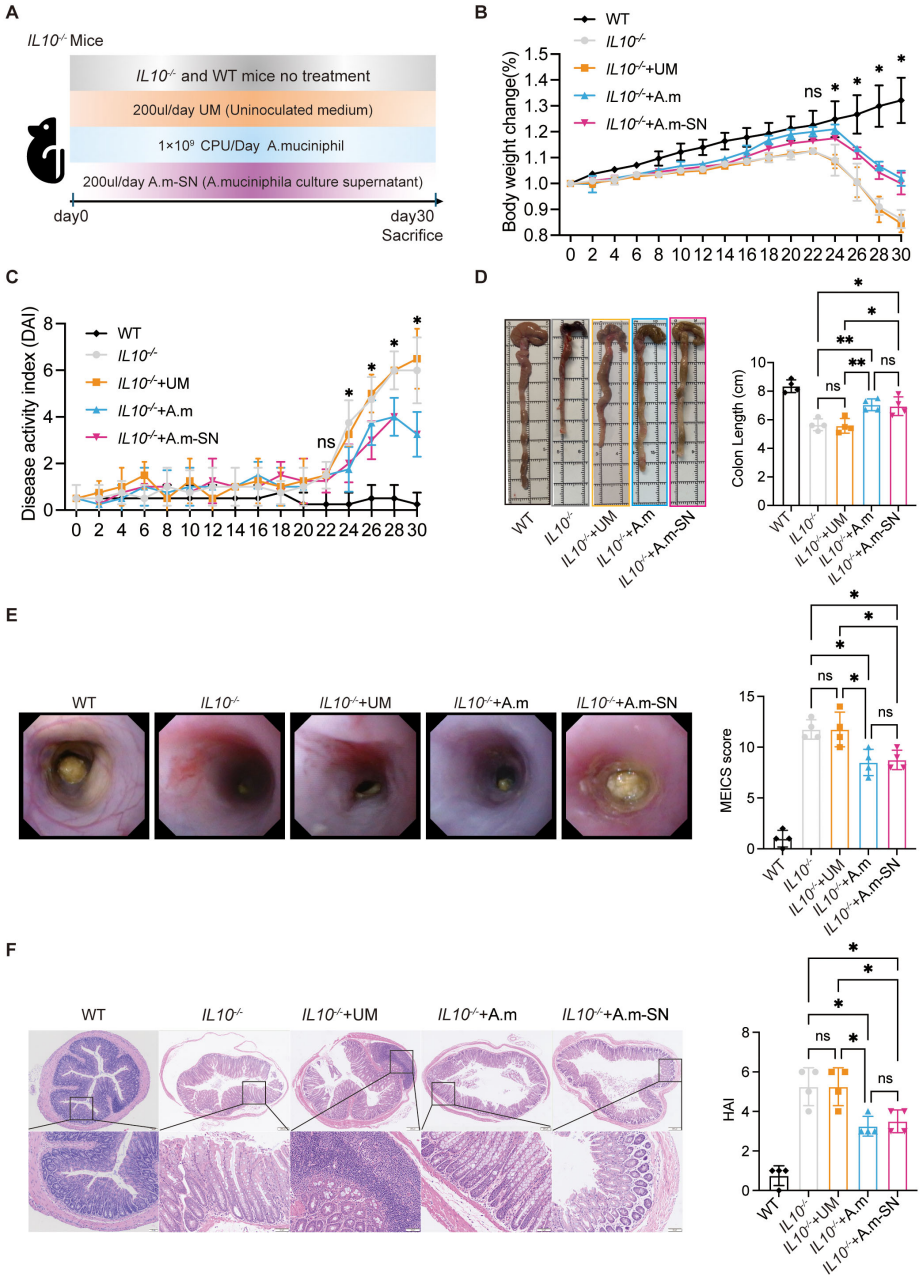
Administration of *A.muciniphila* and its culture supernatant in IL-10 knockout mice. **(A)** Graphical representation showing the process of chronic colitis in IL-10 knockout mice. Mice were divided into *IL10^-/-^*, *IL10^-/-^*+UM, *IL10^-/-^*+A.m and *IL10^-/-^*+A.m-SN groups. **(B)** Body weight changes were daily monitored (day 30: IL-10^−^/^−^ + A.m-SN vs IL-10^−^/^−^ + UM, *p* = 0.0002; IL-10^−^/^−^ + A.m-SN vs IL-10^−^/^−^, *p* = 0.0007; IL10^-/-^+A.m vs IL10^-/-^, *p* = 0.0002; IL10^-/-^+A.m vs IL10^-/-^ + UM, *p* < 0.0001). **(C)** DAI was recorded according to rectal bleeding, diarrhea and body weight change (day 30: IL-10^−^/^−^ + A.m-SN vs IL-10^−^/^−^ + UM, *p* = 0.0095; IL-10^−^/^−^ + A.m-SN vs IL-10^−^/^−^, *p* = 0.0273; IL10^-/-^+A.m vs IL10^-/-^, *p* = 0.0273; IL10^-/-^+A.m vs IL10^-/-^ + UM, *p* = 0.0095). **(D)** Colon lengths were observed and analyzed (IL-10^−^/^−^ + A.m-SN vs IL-10^−^/^−^ + UM, *p* = 0.0101; IL-10^−^/^−^ + A.m-SN vs IL-10^−^/^−^, *p* = 0.0153; IL10^-/-^+A.m vs IL10^-/-^, *p* = 0.0088; IL10^-/-^+A.m vs IL10^-/-^ + UM, *p* = 0.0058). **(E)** Representative colonoscopic images and MEICS scores of IL-10 knockout mice(IL-10^−^/^−^ + A.m-SN vs IL-10^−^/^−^ + UM, *p* = 0.0202; IL-10^−^/^−^ + A.m-SN vs IL-10^−^/^−^, *p* = 0.0202; IL10^-/-^+A.m vs IL10^-/-^, *p* = 0.0114; IL10^-/-^+A.m vs IL10^-/-^ + UM, *p* = 0.0114). **(F)** Representative photomicrographs of HE in colonic tissues and HAI scores (IL-10^−^/^−^ + A.m-SN vs IL-10^−^/^−^ + UM, *p* = 0.0283; IL-10^−^/^−^ + A.m-SN vs IL-10^−^/^−^, *p* = 0.0283; IL10^-/-^+A.m vs IL10^-/-^, *p* = 0.0111; IL10^-/-^+A.m vs IL10^-/-^ + UM, *p* = 0.0111). Data are presented as mean ± SD, *p ≤ 0.05; **p ≤ 0.01; ns, not significant. one-way ANOVA **(B-F)**.

IL-10^−^/^−^ mice are a widely used model for studying intestinal inflammation, as they typically develop spontaneous colitis between 7 and 15 weeks of age. In this study, 5- to 6-week-old IL-10^−^/^−^ mice were randomized into four experimental groups: a control group without intervention (IL-10^−^/^−^), a group that received oral gavage of A.m (IL-10^−^/^−^ + A.m), a group that received oral gavage of A.m culture supernatant containing metabolic products (IL-10^−^/^−^ + A.m-SN), and a group that received oral gavage of uninoculated culture medium (IL-10^−^/^−^ + UM). A WT group without treatment was included as a baseline control. The development of intestinal inflammation was monitored over a 30-day period, during which body weight (**Figure 1A**), stool consistency, and fecal occult blood were documented every other day. During the first 20 days, all groups showed consistent weight gain without significant differences. From day 22 onward, weight gain began to slow, and by day 24, a decline in body weight was noted. Interestingly, the IL-10^−^/^−^ + A.m and IL-10^−^/^−^ + A.m-SN groups exhibited significantly less weight loss compared to the other two groups (**Figure 1B**, day 30: IL-10^−^/^−^ + A.m-SN vs IL-10^−^/^−^ + UM, *p* = 0.0002);. Similarly, DAI scores began to diverge on day 24, with mice in the IL-10^−^/^−^ + A.m and A.m-SN treatment groups exhibited suggestively lower DAI scores in contrast to the other two groups (**Figure 1C**, IL-10^−^/^−^ + A.m-SN vs IL-10^−^/^−^ + UM, *p* = 0.0095). Colon length measurements revealed that mice in the IL-10^−^/^−^ + A.m and IL-10^−^/^−^ + A.m-SN groups had significantly longer colons relative to the other two groups (**Figure 1D**, IL-10^−^/^−^ + A.m-SN vs IL-10^−^/^−^ + UM, *p* = 0.0101). Consistently, both endoscopic and histological assessments showed that mice in the IL-10^−^/^−^ + A.m and IL-10^−^/^−^ + A.m-SN groups exhibited lower endoscopic (**Figure 1E**, IL-10^−^/^−^ + A.m-SN vs IL-10^−^/^−^ + UM, *p* = 0.0202) and Histological Activity Index (HAI) scores (**Figure 1F**, IL-10^−^/^−^ + A.m-SN vs IL-10^−^/^−^ + UM, *p* = 0.0283). Collectively, these results reveal that both *A.muciniphila* and its metabolite-containing culture supernatant effectively alleviate intestinal inflammation in IL-10^−^/^−^ mice.

### 3.2 *A.muciniphila* and its culture supernatant promotes the upregulation of tight junction protein expression in IL-10 knockout mice

**Figure 2:**
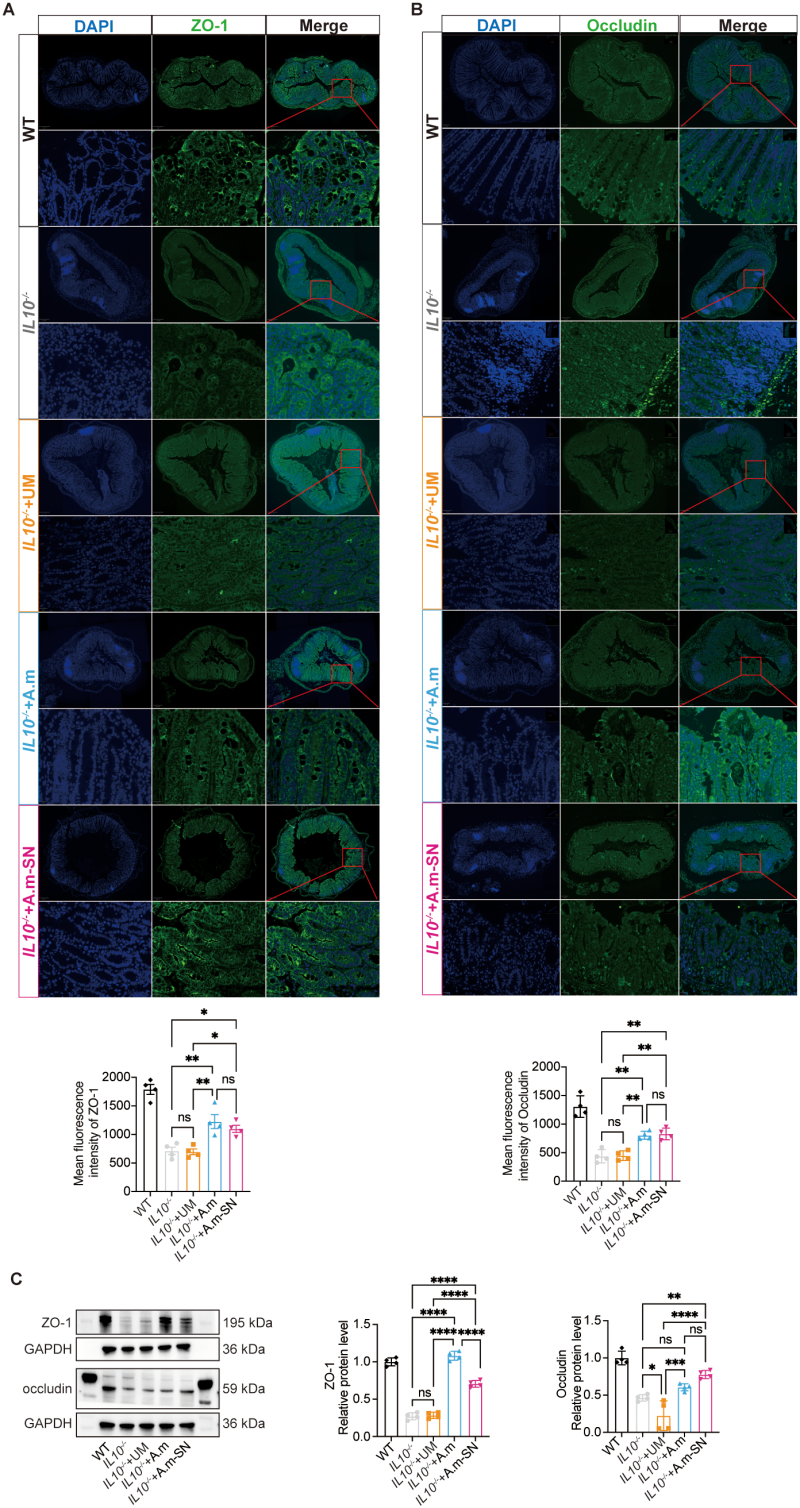
Expression of tight junction proteins ZO-1 and occludin in colonic tissues. **(A)** Immunofluorescence analysis of ZO-1 (green) expression in colonic tissues (IL-10^−^/^−^ + A.m-SN vs IL-10^−^/^−^ + UM, *p* = 0.0231; IL-10^−^/^−^ + A.m-SN vs IL-10^−^/^−^, *p* = 0.00301; IL10^-/-^+A.m vs IL10^-/-^, *p* = 0.0035; IL10^-/-^+A.m vs IL10^-/-^ + UM, *p* = 0.0027). **(B)** Immunofluorescence analysis of occludin (green) expression in colonic tissues (IL-10^−^/^−^ + A.m-SN vs IL-10^−^/^−^ + UM, *p* = 0.032; IL-10^−^/^−^ + A.m-SN vs IL-10^−^/^−^, *p* = 0.0025; IL10^-/-^+A.m vs IL10^-/-^, *p* = 0.0043; IL10^-/-^+A.m vs IL10^-/-^ + UM, *p* = 0.0055). **(C)** Western blot analysis of ZO-1 and occludin in colonic tissues[ZO-1(IL-10^−^/^−^ + A.m-SN vs IL-10^−^/^−^ + UM, *p<*0.0001; IL-10^−^/^−^ + A.m-SN vs IL-10^−^/^−^, *p<*0.0001; IL10^-/-^+A.m vs IL10^-/-^, *p<*0.0001; IL10^-/-^+A.m vs IL10^-/-^ + UM, *p<*0.0001); occludin(IL-10^−^/^−^ + A.m-SN vs IL-10^−^/^−^ + UM, *p<*0.0001; IL-10^−^/^−^ + A.m-SN vs IL-10^−^/^−^, *p* = 0.0056; IL10^-/-^+A.m vs IL10^-/-^, *p* = 0.3517; IL10^-/-^+A.m vs IL10^-/-^ + UM, *p* = 0.001)]Data are presented as mean ± SD, *p ≤ 0.05; **p ≤ 0.01; ***p ≤ 0.001; ****p ≤ 0.0001; ns, not significant. one-way ANOVA **(A-C)**.

To explore in more detail the processes by which *A.muciniphila* and its culture supernatant alleviated intestinal inflammation in IL-10^−^/^−^ mice, immunofluorescence staining was performed on colonic tissue sections. Zonula occludens-1 (ZO-1) and occludin represent critical structural proteins involved in the formation and maintenance of intestinal epithelial tight junctions, which are essential for preserving mucosal barrier integrity. This investigation demonstrated that ZO-1 signal was markedly intensified in mice treated with *A.muciniphila* or its metabolite-containing culture supernatant in contrast with the other two cohorts (**Figure 2A**). Similarly, occludin expression was significantly elevated in the IL-10^−^/^−^ + A.m-SN group relative to both the IL-10^−^/^−^ and IL-10^−^/^−^ + UM groups(**Figure 2B**). Western blot analysis showed that A.m culture supernatant treatment restored the expression of tight junction proteins ZO-1 and occludin in IL-10^−^/^−^ mice (**Figure 2C**). These findings suggest that *A.muciniphila* and its culture supernatant alleviate colitis in IL-10^−^/^−^ mice by upregulating epithelial tight junction proteins ZO-1 and occludin, thus reinforcing the gut barrier.

### 3.3 *A.muciniphila* and its culture supernatant inhibit pro-inflammatory cytokine release while enhancing anti-inflammatory cytokine production

**Figure 3:**
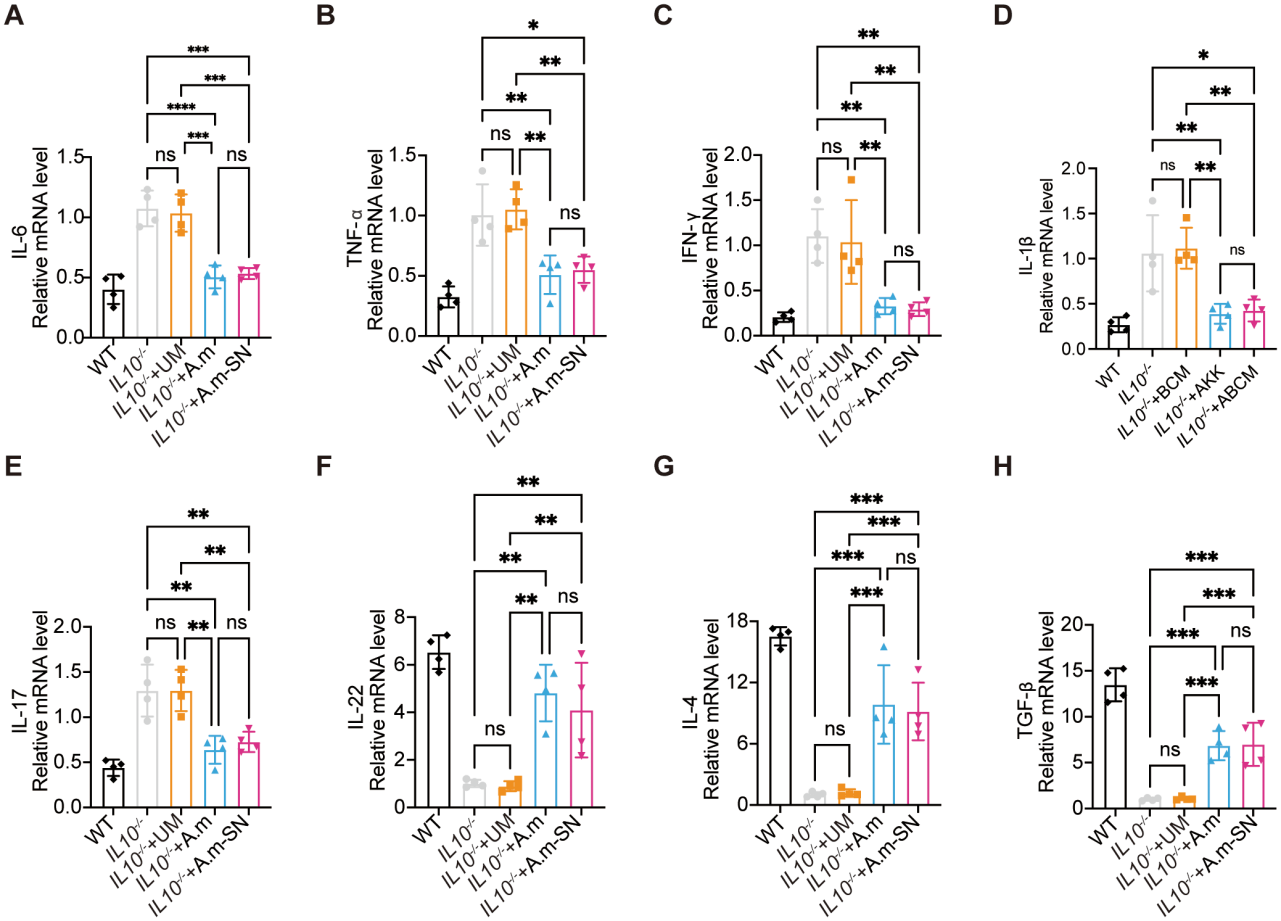
Colonic mRNA expression levels of pro-inflammatory and anti-inflammatory cytokines. **(A)** Quantitative PCR analysis of IL-6 mRNA expression (IL-10^−^/^−^ + A.m-SN vs IL-10^−^/^−^ + UM, *p* = 0.0002; IL-10^−^/^−^ + A.m-SN vs IL-10^−^/^−^, *p* = 0.0002; IL10^-/-^+A.m vs IL10^-/-^, *p* < 0.0001; IL10^-/-^+A.m vs IL10^-/-^ + UM, *p* = 0.0001). **(B)** Quantitative PCR analysis of TNF-α mRNA expression(IL-10^−^/^−^ + A.m-SN vs IL-10^−^/^−^ + UM, *p* = 0.0052; IL-10^−^/^−^ + A.m-SN vs IL-10^−^/^−^, *p* = 0.0113; IL10^-/-^+A.m vs IL10^-/-^, *p* = 0.0057; IL10^-/-^+A.m vs IL10^-/-^ + UM, *p* = 0.0026). **(C)** Quantitative PCR analysis of IFN-γ mRNA expression(IL-10^−^/^−^ + A.m-SN vs IL-10^−^/^−^ + UM, *p* = 0.0063; IL-10^−^/^−^ + A.m-SN vs IL-10^−^/^−^, *p* = 0.0031; IL10^-/-^+A.m vs IL10^-/-^, *p* = 0.0046; IL10^-/-^+A.m vs IL10^-/-^ + UM, *p* = 0.0093). **(D)** Quantitative PCR analysis of IL-1β mRNA expression(IL-10^−^/^−^ + A.m-SN vs IL-10^−^/^−^ + UM, *p* = 0.0054; IL-10^−^/^−^ + A.m-SN vs IL-10^−^/^−^, *p* = 0.0105; IL10^-/-^+A.m vs IL10^-/-^, *p* = 0.0067; IL10^-/-^+A.m vs IL10^-/-^ + UM, *p* = 0.0034). **(E)** Quantitative PCR analysis of IL-17 mRNA expression(IL-10^−^/^−^ + A.m-SN vs IL-10^−^/^−^ + UM, *p* = 0.0054; IL-10^−^/^−^ + A.m-SN vs IL-10^−^/^−^, *p* = 0.0054; IL10^-/-^+A.m vs IL10^-/-^, *p* = 0.0016; IL10^-/-^+A.m vs IL10^-/-^ + UM, *p* = 0.0015). **(F)** Quantitative PCR analysis of IL-22 mRNA expression(IL-10^−^/^−^ + A.m-SN vs IL-10^−^/^−^ + UM, *p* = 0.0065; IL-10^−^/^−^ + A.m-SN vs IL-10^−^/^−^, *p* = 0.0087; IL10^-/-^+A.m vs IL10^-/-^, *p* = 0.0014; IL10^-/-^+A.m vs IL10^-/-^ + UM, *p* = 0.0011). **(G)** Quantitative PCR analysis of IL-4 mRNA expression(IL-10^−^/^−^ + A.m-SN vs IL-10^−^/^−^ + UM, *p* = 0.0009; IL-10^−^/^−^ + A.m-SN vs IL-10^−^/^−^, *p* = 0.0007; IL10^-/-^+A.m vs IL10^-/-^, *p* = 0.0003; IL10^-/-^+A.m vs IL10^-/-^ + UM, *p* = 0.0004). **(H)** Quantitative PCR analysis of TGF-β mRNA expression (IL-10^−^/^−^ + A.m-SN vs IL-10^−^/^−^ + UM, *p* = 0.0005; IL-10^−^/^−^ + A.m-SN vs IL-10^−^/^−^, *p* = 0.0004; IL10^-/-^+A.m vs IL10^-/-^, *p* = 0.0005; IL10^-/-^+A.m vs IL10^-/-^ + UM, *p* = 0.0006). Data are presented as mean ± SD, *p ≤ 0.05; **p ≤ 0.01; ***p ≤ 0.001; ****p ≤ 0.0001; ns, not significant. one-way ANOVA **(A-H)**.

Following the observation that *A.muciniphila* and its culture supernatant enhanced epithelial tight junction protein expression, we next explored their effects on mucosal immune responses, particularly the modulation of pro- and anti-inflammatory cytokine synthesis. mRNA levels of the pro-inflammatory mediators TNF-α, IL-6, IL-17, IL-1β, and IFN-γ were significantly reduced in the IL-10^−^/^−^ + A.m and IL-10^−^/^−^ + A.m-SN groups compared with the IL-10^−^/^−^ and IL-10^−^/^−^ + UM groups (**Figures 3A–E**). Conversely, the anti-inflammatory cytokines IL-4, TGF-β, and IL-22 were significantly upregulated in the IL-10^−^/^−^ + A.m and IL-10^−^/^−^ + A.m-SN groups relative to the other two groups (**Figures 3F, G**).

### 3.4 *A.muciniphila* and its culture supernatant improve gut microbiota dysbiosis in IL-10 knockout mice

**Figure 4:**
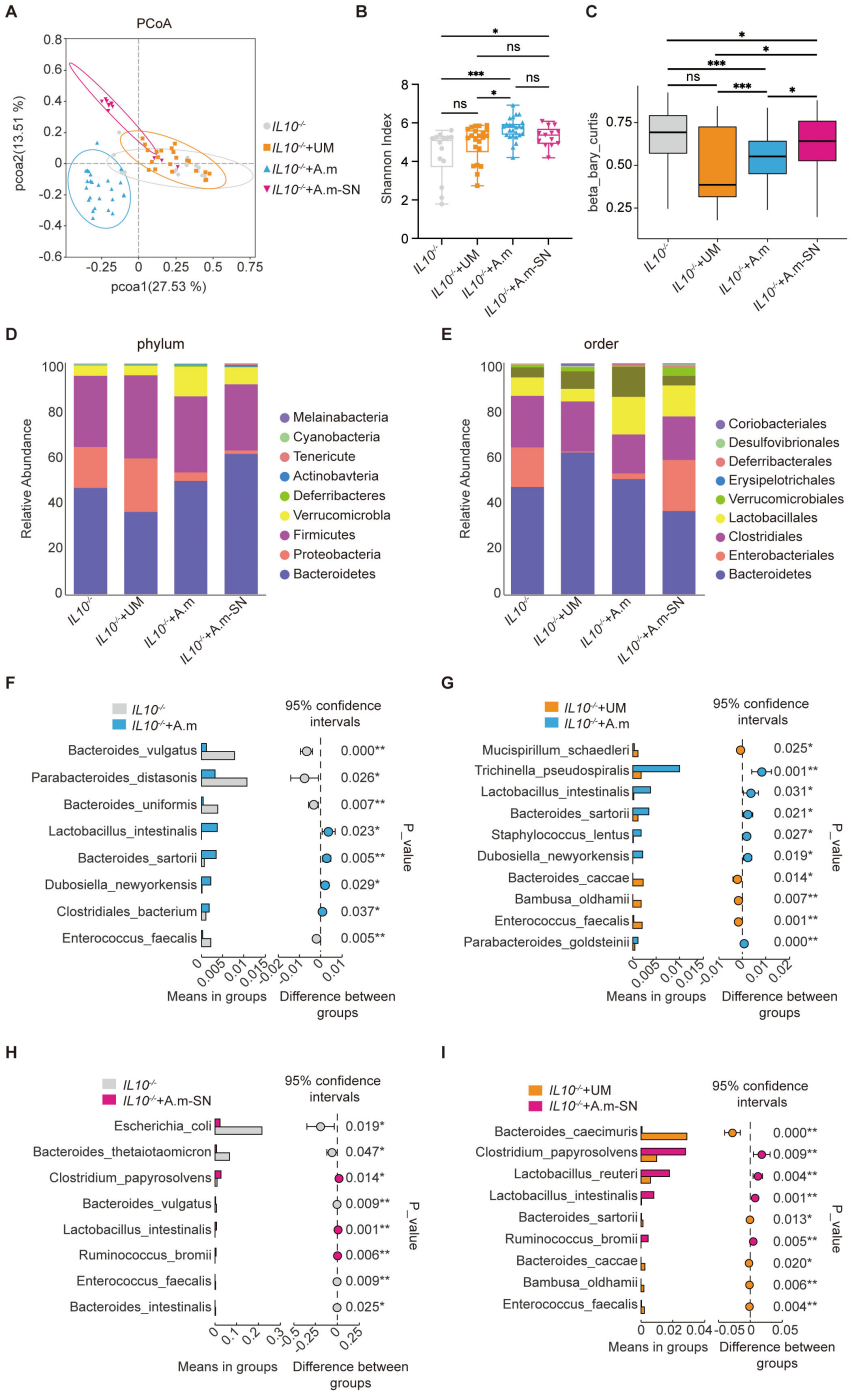
Gut microbiota analysis in IL-10^−^/^−^ mice following treatment with *A.muciniphila* or its culture supernatant. **(A)** Principal coordinate analysis (PCoA) showing distinct clustering between groups. **(B)** Shannon index indicating differences in alpha diversity. **(C)** Beta diversity based on Bray–Curtis distance. **(D)** Relative abundance at the phylum level. **(E)** Relative abundance at the order level. **(F–I)** Species-level comparisons showing differential abundance among groups. IL-10^−^/^−^ (n = 14), IL-10^−^/^−^ + UM (n = 22), IL-10^−^/^−^ + A.m (n = 25), and IL-10^−^/^−^ + A.m-SN (n = 13).

To further assess the role of *A.muciniphila* and its culture supernatant on the gut microbial ecosystem, we collected fecal samples from all four groups of mice and performed 16S rDNA sequencing. This analysis aimed to characterize the microbial composition and diversity, and to determine whether *A muciniphila* or its culture supernatant could restore microbial balance in IL-10^−^/^−^ mice with colitis. Principal Coordinates Analysis (PCoA) based on 16S rDNA sequencing showed distinct clustering of gut microbiota profiles amongst the four groups. Mice in the IL-10^−^/^−^ and IL-10^−^/^−^ + UM groups exhibited similar microbial communities, with overlapping distributions, indicating persistent dysbiosis. In contrast, the IL-10^−^/^−^ + A.m and IL-10^−^/^−^ + A.m-SN groups formed separate and clearly distinct clusters, suggesting that both *A.muciniphila* and its culture supernatant significantly altered and partially restored the gut microbial composition (**Figure 4A**). As revealed in the Shannon index (**Figure 4B**), mice in the IL-10^−^/^−^ + A.m and IL-10^−^/^−^ + A.m-SN groups exhibited higher alpha diversity compared to the IL-10^−^/^−^ and IL-10^−^/^−^ + UM groups (IL10^-/-^ vs. IL10^-/-^+A.m, *p* = 0.0353; IL-10^−^/^−^ + A.m-SN vs IL-10^−^/^−^ + UM, *p* = 0.014), indicating a more complex and balanced microbial community. Beta diversity analysis using Bray–Curtis dissimilarity (**Figure 4C**, IL10^-/-^ vs. IL10^-/-^+A.m, *p* = 0.0353; IL-10^−^/^−^ + A.m-SN vs IL-10^−^/^−^ + UM, *p* = 0.014) showed significant shifts in microbial composition among the groups. The IL-10^−^/^−^ + A.m group, in particular, displayed reduced intra-group variability and greater separation from the IL-10^−^/^−^ and IL-10^−^/^−^ + UM groups, suggesting that *A.muciniphila* intervention led to a more stable and distinct microbial profile. A.m-SN treatment also resulted in significant compositional changes, though with slightly higher variability. The analysis of relative species abundance showed that at the phylum level (**Figure 4D**), IL-10^−^/^−^ and IL-10^−^/^−^ + UM mice demonstrated a greater relative abundance of *Proteobacteria* and worse levels of *Bacteroidetes* and *Verrucomicrobia*, indicating microbial dysbiosis. In contrast, mice treated with *A.muciniphila* or its culture supernatant (A.m and A.m-SN groups) showed an increased proportion of *Bacteroidetes* and *Verrucomicrobia*, along with a marked reduction in Proteobacteria, suggesting a partial restoration of microbial balance. At the order level (**Figure 4E**), the IL-10^−^/^−^ + A.m and A.m-SN groups demonstrated higher relative abundance of *Bacteroidales* and *Verrucomicrobiales*, with reduced levels of *Enterobacteriales*, an order often associated with intestinal inflammation. Additionally, enrichment of beneficial taxa such as *Lactobacillales* and *Clostridiales* was observed in the *A.muciniphila*-treated groups compared to controls. Differential species-level analysis revealed significant alterations in gut microbiota composition following treatment with *A.muciniphila* or its culture supernatant in IL-10^−^/^−^ mice. Compared with the IL-10^−^/^−^ group (**Figure 4F**), mice treated with *A.muciniphila* showed increased relative abundance of beneficial bacterial species such as *Parabacteroides distasonis*, *Lactobacillus intestinalis*, and *Dubosiella newyorkensis*, while potentially pathogenic species including *Bacteroides vulgatus* and *Enterococcus faecalis* were significantly reduced. When compared to the IL-10^−^/^−^ + UM group (**Figure 4G**), the IL-10^−^/^−^ + A.m group demonstrated enrichment of species such as *Mucispirillum schaedleri*, *Dubosiella newyorkensis*, and *Lactobacillus intestinalis*, alongside a marked decrease in *Enterococcus faecalis* and *Parabacteroides goldsteinii*. Similarly, the IL-10^−^/^−^ + A.m-SN group exhibited decreased abundance of pro-inflammatory species like *Escherichia coli* and *Enterococcus faecalis*, and increased abundance of anti-inflammatory or beneficial taxa such as *Ruminococcus bromii*, *Lactobacillus intestinalis*, and *Clostridium papyrosolvens* compared to the IL-10^−^/^−^ group (**Figure 4H**). Compared to the IL-10^−^/^−^ + UM group (**Figure 4I**), the A.m-SN treatment significantly enriched species including *Lactobacillus reuteri*, *Ruminococcus bromii*, and *Clostridium papyrosolvens*, while reducing the abundance of *Enterococcus faecalis* and *Bacteroides caccae*. These compositional shifts indicate that both *A.muciniphila* and its culture supernatant modulate specific microbial species associated with either pro-inflammatory or anti-inflammatory effects, contributing to the restoration of microbial homeostasis in colitis.

### 3.5 *A.muciniphila* alters the metabolic profile of the culture medium and produces potentially bioactive culture supernatant

**Figure 5:**
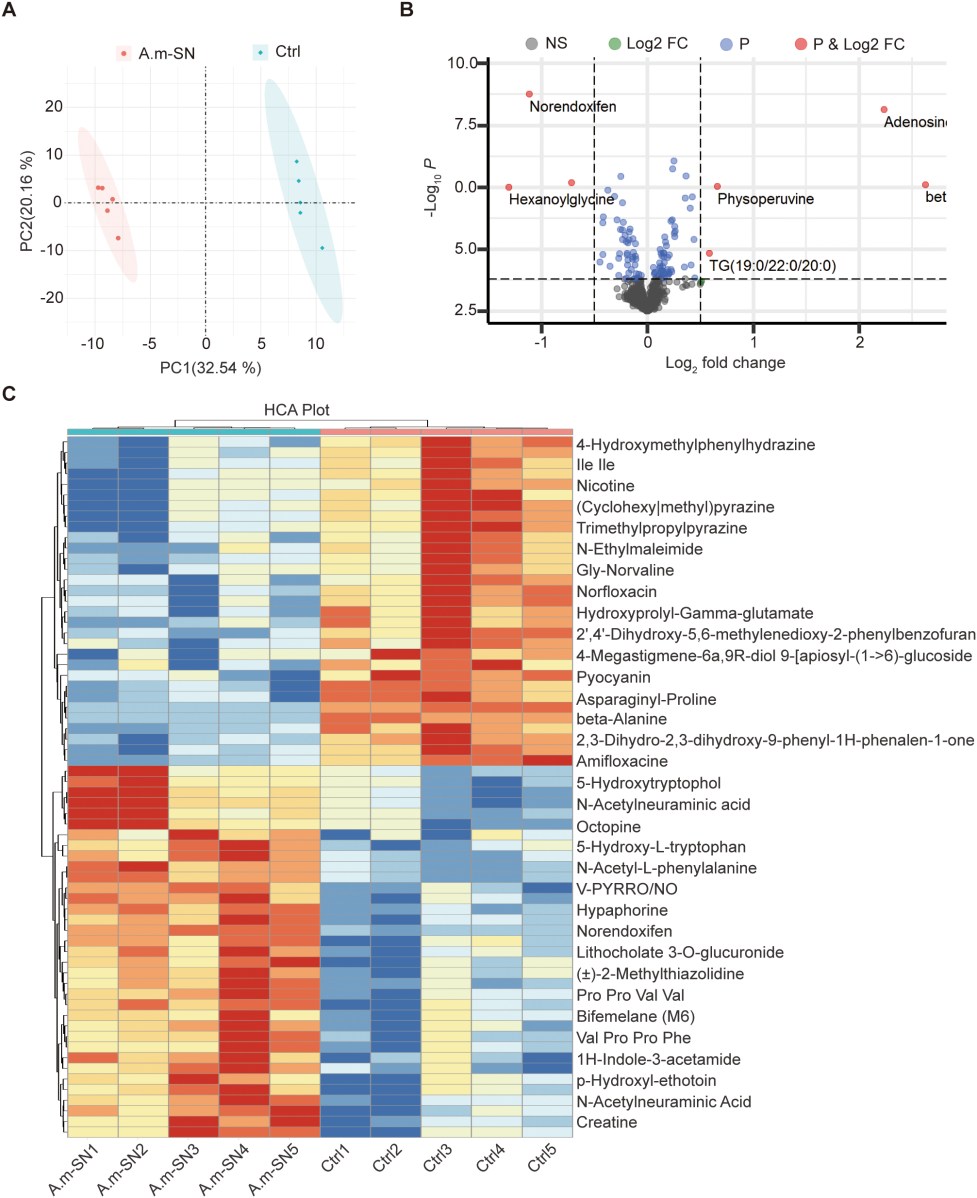
Untargeted metabolomic profiling of *A.muciniphila* culture supernatants. **(A)** Principal component analysis (PCA) showing a clear separation of metabolomic profiles between *A.muciniphila*-conditioned medium (A.m-SN) and uninoculated control medium (Ctrl). **(B)** Volcano plot displaying differentially abundant culture supernatant between the two groups. Red dots represent significantly upregulated culture supernatant in the A.m-SN group (|log_2_FC| > 1, *P* < 0.05). **(C)** Heatmap of the top differential culture supernatant identified by hierarchical clustering analysis. Rows represent individual culture supernatant, and columns represent biological replicates.

To assess the impact of A. muciniphila on the metabolic composition of the culture medium, we performed untargeted metabolomic analysis of both A.muciniphila-conditioned medium (A.m-SN) and uninoculated control medium (Ctrl). Principal component analysis (PCA) demonstrated clear group divergence implying that *A.muciniphila* substantially altered the overall metabolite structure of the medium (**Figure 5A**). Volcano plot analysis further identified a series of culture supernatant that were significantly upregulated in the A.m-SN group, including adenosine, norendoxifen, hexanoylglycine, and physoperuvine (**Figure 5B**). These compounds have been previously associated with immunomodulatory, anti-inflammatory, and epithelial-protective effects. For instance, adenosine plays a well-known role in immune suppression through adenosine receptor signaling, while betaine is involved in methylation balance and osmoprotection. Hierarchical clustering analysis further supported these differences, showing a distinct metabolite expression pattern between the A.m-SN and Ctrl groups (**Figure 5C**). In particular, *A.muciniphila*-conditioned medium was enriched for several amino acid derivatives (e.g., 5-hydroxytryptophol, 5-hydroxy-L-tryptophan), nucleotide-related culture supernatant (e.g., adenosine), and microbial-associated culture supernatant (e.g., N-acetylneuraminic acid, hypaphorine, lithocholate derivatives). These results demonstrate that *A. muciniphila* substantially reprograms the metabolic composition of the culture medium, and the identified culture supernatant may provide candidate molecules for future functional validation.

## 4 Discussion

In this study, we investigated the protective effects of the culture supernatant of A.m-SN in IL-10^−^/^−^ mice, a well-established model of spontaneous chronic colitis. A.m-SN treatment significantly alleviated intestinal inflammation, as evidenced by improved body weight maintenance, reduced DAI scores, elongated colons, and ameliorated histological and endoscopic changes. Similar benefits were observed in the group receiving live A.m, consistent with its reported barrier- and microbiota-modulating effects. Notably, the efficacy of A.m-SN alone indicates that soluble factors secreted by A.m are sufficient to confer robust protection in this model, even without the presence of live bacteria. Mechanistically, A.m-SN suppressed pro-inflammatory cytokines (tumor necrosis factor-α [TNF-α], IL-6, IL-17A, interferon-γ [IFN-γ]) while enhancing anti-inflammatory mediators (IL-4, transforming growth factor-β [TGF-β], IL-22), indicating a shift toward an anti-inflammatory immune milieu. Consistent with these immune changes, A.m-SN also upregulated epithelial tight junction proteins, thereby reinforcing barrier function. Moreover, untargeted metabolomic profiling identified distinct bioactive metabolites, including adenosine and tryptophan derivatives, as candidate molecules that may contribute to these protective effects.

The IL-10^−^/^−^ mouse is a widely used model of chronic, microbiota-driven colitis that shares key features with Crohn’s disease (36–39). Because disease development depends strongly on gut microbiota, it is well suited for studies of host–microbe interactions and microbiota-based therapies (40, 41). Previous studies have shown that dietary polysaccharides can enrich A.m in IL-10^−^/^−^ mice, indirectly improving mucus barrier function and reducing inflammation (42). Moreover, colonization with A.m strain ATCC BAA-835 did not worsen colitis, supporting its safety even in genetically susceptible hosts (43). However, these studies primarily relied on indirect modulation of A.m. In contrast, we directly compared live bacteria with their culture supernatant in IL-10^−^/^−^ mice to clarify how A.m and its secreted products regulate immunity and barrier integrity.

In our study, supplementation with A.m or its metabolite-containing supernatant significantly reshaped the gut microbial community in IL-10^−^/^−^ mice, characterized by an increased abundance of beneficial taxa and a reduced prevalence of putative pathobionts. These compositional changes were accompanied by improved alpha diversity and distinct clustering in beta diversity analyses, indicating a robust microbiota-remodeling effect. Importantly, these microbial shifts also occurred in the absence of live bacterial colonization, as observed in the metabolite-only treatment group, suggesting that bioactive compounds secreted by A.m play a critical role in mediating these effects. These findings align with previous evidence that enrichment of A.m correlates with enhanced intestinal barrier function and reduced inflammatory burden (42). Notably, our observation that A.m-derived culture supernatant alone could induce comparable microbial shifts suggests that certain bioactive compounds can mediate these effects independently of live bacterial colonization. Such culture supernatant, potentially containing short-chain fatty acids and other signaling molecules, may influence community dynamics through cross-feeding interactions and competitive exclusion of pathobionts (44). Moreover, emerging evidence indicates that the inflammatory microenvironment, together with bacteriophage activity, can modulate microbial gene expression via deoxyribonucleic acid inversion mechanisms, thereby altering bacterial metabolic capacity and immune-modulatory functions (45). Gut microbial composition and metabolite profiles have also been increasingly recognized as predictors of therapeutic responsiveness in IBD, underscoring their clinical relevance as biomarkers and targets for microbiota-based interventions (46). Furthermore, modulation of the gut microbiota may attenuate mucosal inflammation indirectly by regulating innate immune effectors such as neutrophils, whose recruitment and activation are tightly controlled by microbial signals (47). Collectively, the microbiota remodeling observed in our study is likely to act synergistically with the direct epithelial and immunomodulatory effects of A.m, thereby contributing to the attenuation of mucosal inflammation and the restoration of immune homeostasis in IL-10^−^/^−^ colitis.

Given that restoration of a balanced gut microbiota is closely linked to improved epithelial barrier integrity, we further examined whether A.m and its culture supernatant could modulate tight junction protein levels in IL-10^−^/^−^ mice. Tight junction proteins, such as occludin and ZO-1, regulate intestinal permeability and prevent antigen and pathogen translocation (48). In IBD, reduced expression or mislocalization of these proteins contributes to increased permeability and persistent mucosal inflammation. In our study, immunofluorescence staining demonstrated that supplementation with A.m or its metabolite-containing supernatant significantly upregulated ZO-1 expression in colonic epithelial cells compared with untreated IL-10^−^/^−^ mice. Occludin expression was also increased, particularly in the A.m-treated group, with the metabolite-supplemented group showing a positive but non-significant trend. These findings align with those of earlier studies showing that A.m, either alone or in combination with bioactive metabolites such as inosine, enhances tight junction integrity and mucus layer thickness, potentially through host signaling pathways including AMPK and TLR2, or by producing metabolites that serve as substrates or regulators of epithelial cell function (49). Similar effects have been reported with plant-derived bioactives, such as *Artemisia argyi* polyphenols, which restore microbial balance and upregulate tight junction proteins to attenuate experimental colitis (50). Furthermore, mechanistic insights from mucosal repair research show that while ZO-1 may not be essential for basal epithelial integrity, it is critical for effective restitution following injury, underscoring its therapeutic relevance in chronic inflammation (51). The preservation and enhancement of tight junction protein expression observed here are therefore likely to synergize with microbiota modulation, reducing intestinal permeability, limiting immune activation, and interrupting the cycle of inflammation and barrier disruption characteristic of IBD. Notably, the finding that metabolite supplementation alone could induce similar effects highlights the potential of identifying and harnessing specific A.m-derived compounds as postbiotic interventions for barrier restoration in chronic colitis.

In addition to enhancing epithelial barrier integrity, A.m and its culture supernatant exerted pronounced immunomodulatory effects in IL-10^−^/^−^ mice, reflected by alterations in key pro- and anti-inflammatory cytokines within colonic tissue. Chronic intestinal inflammation in IBD is driven in part by elevated pro-inflammatory cytokines such as TNF-α, IL-6, IL-17, IL-1β, and IFN-γ, which collectively promote leukocyte recruitment, amplify epithelial injury, and sustain a feed-forward inflammatory loop (52, 53). Conversely, anti-inflammatory cytokines, including IL-4, TGF-β, and IL-22, support immune regulation, epithelial repair, and mucosal homeostasis (54, 55). Our results revealed that supplementation with A.m or its metabolite-containing supernatant significantly downregulated messenger ribonucleic acid levels of TNF-α, IL-6, IL-17, IL-1β, and IFN-γ, while concurrently upregulating IL-4, TGF-β, and IL-22 compared with untreated IL-10^−^/^−^ mice. These shifts indicate restoration of the pro-/anti-inflammatory balance, favoring an environment conducive to mucosal healing and immune tolerance. Mechanistically, this dual action—suppressing pro-inflammatory mediators while enhancing anti-inflammatory signals—may occur through direct interactions of A.m-derived molecules with pattern recognition receptors or indirectly via microbiota reshaping that influences immune cell activation. Such immune reprogramming is particularly relevant in IL-10^−^/^−^ mice, where defective IL-10 signaling skews mucosal immunity toward chronic inflammation. By modulating both innate and adaptive immune responses, A.m and its culture supernatant may compensate for the absence of IL-10, mitigating T cell-driven pathology and breaking the cycle of barrier disruption and microbial translocation. The observation that metabolite supplementation alone elicited comparable immunomodulatory effects further supports the feasibility of developing postbiotic-based strategies for chronic colitis.

Building on these immunological findings, we further sought to identify components of the culture supernatant responsible for the protective effects of A.m in IL-10^−^/^−^ mice. Untargeted metabolomic profiling of the A.m-SN revealed distinct alterations compared with unconditioned medium, characterized by significant enrichment of multiple bioactive compounds. Clear separation of groups in principal component analysis, together with concordant volcano plots and heatmaps, indicates that A.m broadly reprogrammed the small-molecule composition of the medium rather than simply shifting a few abundant species. Several metabolite classes emerged that plausibly converge on epithelial protection and anti-inflammatory effects: purine nucleosides (adenosine), amino-acid/tryptophan–linked derivatives (e.g., 5-hydroxy-L-tryptophan, 5-hydroxytryptophol, hypaphorine), methyl-donor/osmoprotective molecules (betaine), bile acid–like features (lithocholate-related), and host-glycan–associated moieties (e.g., sialic acid–related annotations). These findings support a model in which A.m imprints a bioactive metabolite signature capable of modulating mucosal immunity and barrier integrity. Among the candidates, adenosine provides the most direct mechanistic link between the A.m metabolome and immune suppression. Extracellular adenosine signals via A2A and A2B receptors to restrain pro-inflammatory cytokine production, promote regulatory T-cell programs, and bias cellular metabolism toward oxidative phosphorylation (56, 57). Therefore, enrichment of adenosine in A.m-SN represents a credible pathway by which the conditioned medium could dampen inflammation and promote epithelial homeostasis. Tryptophan-pathway derivatives and the indole-type hypaphorine suggest a complementary axis of host–microbe crosstalk. The specific metabolites detected here align more closely with serotonin-pathway intermediates than canonical aryl hydrocarbon receptor ligands; however, tryptophan-derived culture supernatants are known to influence epithelial tight junctions, enteroendocrine signaling, and innate immune calibration (58, 59). In this context, A.m-derived or -modified tryptophan metabolites may fine-tune epithelial and myeloid responses, potentially acting synergistically with adenosine to reinforce an anti-inflammatory set point. Overall, these findings position A.m not only as a live biotherapeutic candidate but also as a source of postbiotic culture supernatant with defined mechanisms of action. Among the candidate metabolites, adenosine and tryptophan-related derivatives are particularly noteworthy given their established roles in immune suppression and epithelial protection. Our results suggest that these culture supernatants may contribute to the beneficial effects of A.m-SN; however, this conclusion remains hypothesis-generating. Direct functional validation—such as testing purified metabolites, supplementation studies, or receptor blockade—will be necessary to establish causality. From a translational perspective, targeting specific metabolites such as adenosine may provide safer and more controlled interventions, particularly for patients in whom live bacterial administration poses safety concerns. Concurrently, integrating microbial modulation with metabolite-based strategies could offer a multi-layered approach to restoring mucosal homeostasis in chronic inflammatory conditions such as IBD. Our study has certain limitations: the identified metabolites have not yet been individually validated, and their direct contribution to colitis improvement remain to be demonstrated. Therefore, our work should be considered an important first step—providing candidate leads and a roadmap for future mechanistic and translational studies, rather than definitive proof of mechanism.

In conclusion, we demonstrate that the culture supernatant of A.m alleviated colitis in IL-10^−^/^−^ mice by suppressing pro-inflammatory cytokines, enhancing anti-inflammatory mediators, and upregulating epithelial tight junction proteins ZO-1 and occludin. Untargeted metabolomic profiling identified candidate bioactive metabolites, including adenosine and tryptophan derivatives, that may contribute to these protective effects. The functional roles of individual metabolites require validation in future targeted studies; however, our findings provide evidence that A.m–derived culture supernatant represents a promising postbiotic strategy for IBD management.

## Data Availability

The original contributions presented in the study are publicly available. This data can be found here: SRA database / BioProject PRJNA1310766.

## Supplementary material

Supplementary Figure 1 Oral gavage does not alter spontaneous colitis severity in Il10^−^/^−^ mice. **(A)** Body weight changes were daily monitored. **(B)** DAI was recorded according to rectal bleeding, diarrhea and body weight change. **(C)** Colon lengths were observed and analyzed. **(D)** Representative colonoscopic images and MEICS scores of IL-10 knockout mice. **(E)** Representative photomicrographs of HE in colonic tissues and HAI scores. Data are presented as mean ± SD; ns, not significant. Mann–Whitney U test.
