# Supplementary figures and images for: Akkermansia muciniphila and its culture supernatant ameliorate colitis in interleukin-10 knockout mice via gut barrier and immune modulation
Source: Front Immunol. 2025 Nov 17;16:1693007. doi: 10.3389/fimmu.2025.1693007 (PMC12665751; doi:10.3389/fimmu.2025.1693007)

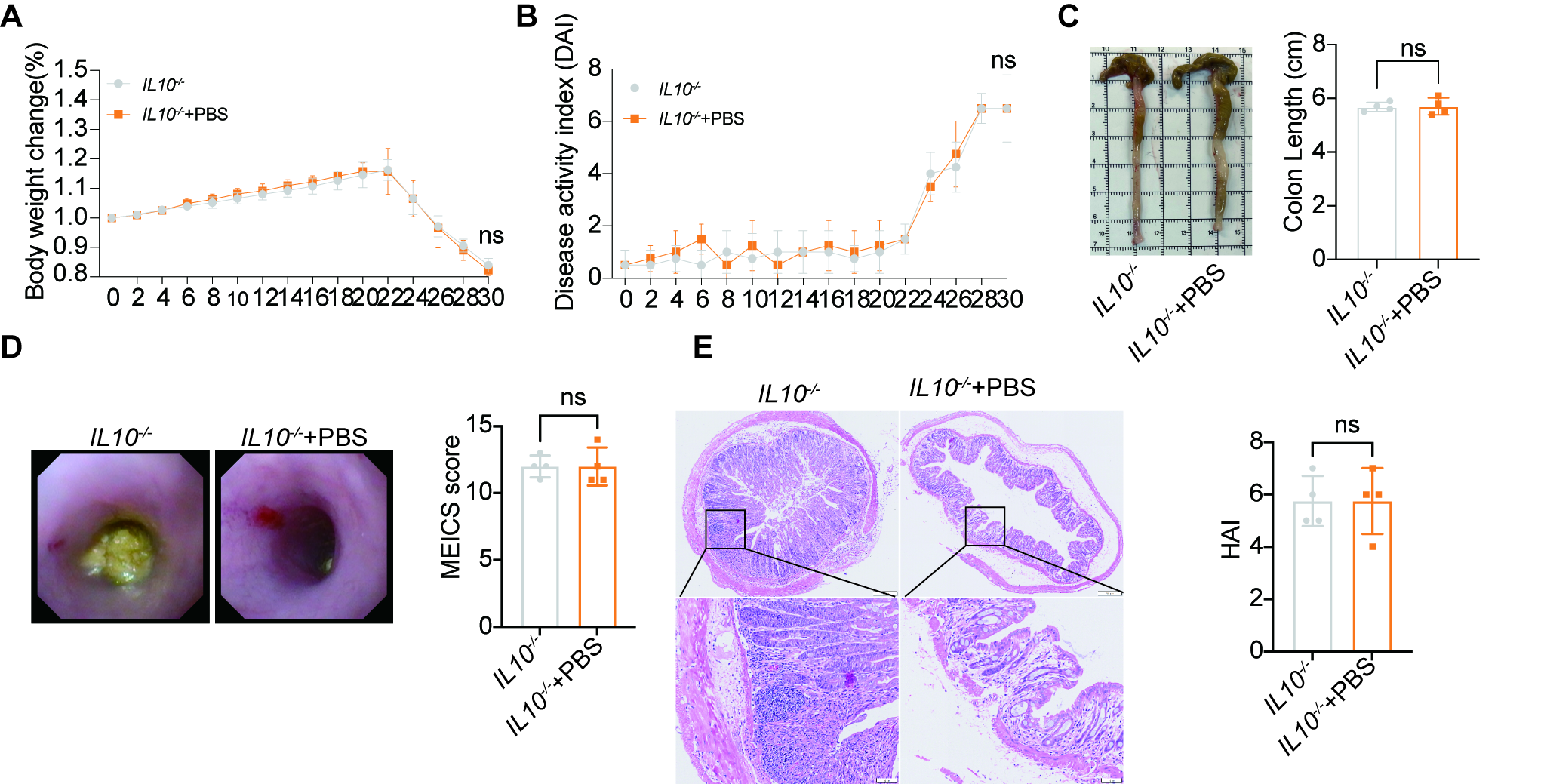

Supplement: Supplementary Figure 1 — Oral gavage does not alter spontaneous colitis severity in Il10−/− mice. (A) Body weight changes were daily monitored. (B) DAI was recorded according to rectal bleeding, diarrhea and body weight change. (C) Colon lengths were observed and analyzed. (D) Representative colonoscopic images and MEICS scores of IL-10 knockout mice. (E) Representative photomicrographs of HE in colonic tissues and HAI scores. Data are presented as mean ± SD; ns, not significant. Mann–Whitney U test. [file Image1.tif]
